# *In vivo* detection of HER2-positive breast cancer and sentinel lymph node metastasis using activatable ICG-conjugated Trastuzumab

**DOI:** 10.3389/fonc.2025.1747065

**Published:** 2026-01-14

**Authors:** Yunpeng Liu, Mahendra Isa, Takahito Nakajima

**Affiliations:** 1 Department of Diagnostic and Interventional Radiology, University of Tsukuba, Tsukuba, Ibaraki, Japan; 2 Center for Cyber Medicine Research, University of Tsukuba, Tsukuba, Ibaraki, Japan

**Keywords:** breast cancer, HER2, indocyanine green, metastasis detection, near-infrared fluorescence, sentinel lymph node, Trastuzumab

## Abstract

Breast cancer continues to pose a significant health threat to women globally, with sentinel lymph node (SLN) metastasis playing a crucial role in treatment planning and prognosis. Conventional diagnostic techniques are invasive, lack dynamic capability, and have limited specificity. Moreover, detecting intraductal spread or microinvasion remains a challenge. Here, we utilized an activatable (quenched) fluorescence probe by conjugating indocyanine green (ICG) with the anti-HER2 antibody Trastuzumab for non-invasive detection of HER2-positive breast cancer and its sentinel lymph node metastasis. Specifically, we conjugated ICG-sulfo-EG4-OSu to Trastuzumab and purified it to obtain Trastuzumab–ICG. *In vitro* live-cell imaging and flow cytometry demonstrated that the conjugate bound specifically to HCC-1419-Luc (HER2-positive) cells and emitted fluorescence, whereas no fluorescence was observed in BT-20-Luc (HER2-negative) cells. Orthotopic tumor models and lymph node metastasis models further confirmed its *in vivo* specificity. These results indicate the potential of Trastuzumab–ICG for high-specificity detection of HER2-positive breast cancer and its lymph node metastases, offering a promising tool for high-contrast tumor visualization and personalized treatment guidance.

## Introduction

Breast cancer is among the most common malignancies worldwide, with over 2.3 million new cases annually (1–3). Although multidisciplinary, biomarker-driven treatments integrating surgery, radiotherapy, and systemic therapy have advanced outcomes, metastasis remains the major cause of mortality, accounting for most breast cancer–related deaths (4–6). Thus, early and accurate detection of metastasis is critical for guiding treatment strategies (7, 8).

Sentinel lymph node (SLN) biopsy plays a central role in staging and surgical decision-making in early breast cancer, as it identifies the first lymph node in the tumor lymphatic drainage pathway. It is the clinical gold standard for axillary assessment and is commonly used in combination with rapid intraoperative pathology, including frozen section analysis and one-step nucleic acid amplification (OSNA). While SLN biopsy helps avoid unnecessary axillary lymph node dissection (ALND), it remains an invasive procedure and may cause complications such as seroma formation and lymphedema (9), affecting patient quality of life. Clinically, SLN biopsy carries a false-negative rate of approximately 5–10%, and widely used blue dyes for SLN mapping (e.g., isosulfan blue, patent blue) are associated with allergic reactions—including anaphylaxis—at reported rates of 0.07% to 2.7% (10). These limitations, combined with the inability of SLN biopsy to provide real-time functional information or reliably detect intraductal spread or microinvasion (11, 12), underscore the need for a non-invasive, real-time intraoperative imaging strategy to improve nodal assessment.

Near-infrared (NIR) fluorescence imaging has emerged as a valuable intraoperative navigation tool, offering superior tissue penetration and high contrast (13, 14). ICG is widely used (15, 16), however, as an ‘always-on’ dye, it suffers from nonspecific accumulation and background fluorescence, which limits tumor-to-background ratios.

To overcome this limitation, we developed an activatable (quenched) fluorescence probe by conjugating ICG to the anti-HER2 antibody Trastuzumab (17, 18). This probe remains quenched (‘off’) in circulation, minimizing background signals, and becomes fluorescent (‘on’) only after binding specifically to HER2, cellular internalization, and lysosomal degradation. As a result, only HER2-expressing tumor cells emit strong fluorescence, enabling precise discrimination from normal tissue (19).

Such activatable antibody-fluorophore conjugates typically rely on dye-dye proximity to maintain a quenched state in circulation, primarily through H-aggregate formation and Förster resonance energy transfer (FRET)-mediated energy dissipation (20, 21). After binding to HER2 on the cell surface, the antibody-receptor complex undergoes internalization via HER2-mediated endocytosis and is trafficked through endosomal compartments to lysosomes (22). The acidic and proteolytic lysosomal environment disrupts dye aggregation and separates the fluorophores from the antibody scaffold, thereby restoring fluorescence (20, 23). This target-dependent activation mechanism provides markedly higher tumor-to-background contrast than conventional ‘always-on’ antibody-fluorophore conjugates.

In this study, we designed and evaluated the Trastuzumab–ICG probe both *in vitro* and *in vivo*, demonstrating its potential for high-contrast detection of HER2-positive breast cancer and sentinel lymph node metastases.

## Materials and methods

### Reagents

ICG-sulfo-EG4-OSu was purchased from AAT Bioquest (Pleasanton, CA, USA). Trastuzumab, a humanized monoclonal antibody targeting HER2, was obtained from Chugai Pharmaceutical Co., Ltd. (Tokyo, Japan). All other chemicals and reagents used were of analytical or reagent grade.

### Synthesis of ICG-conjugated antibody (activatable probe)

As the main objective of this study, we generated an activatable probe (quenched conjugate with on–off capability) by conjugating ICG to Trastuzumab. Trastuzumab (1 mg, 6.8 nmol) and ICG-sulfo-EG4-OSu (54.4 nmol, molar ratio 1:8) were incubated in 0.1 mol/L Na_2_HPO_4_ buffer (pH 8.5) at room temperature for 30 minutes. The reaction mixture was purified using a Sephadex G-50 column (PD-10; GE Healthcare). The concentration of ICG was determined by UV–visible absorption spectroscopy to estimate the labeling efficiency.

The degree of labeling (ICG per antibody) was determined from the UV–visible absorbance spectrum using the instrument’s analysis software, which automatically calculates the dye-to-protein ratio based on absorbance at 800 nm and 280 nm. The purified Trastuzumab–ICG conjugate showed an average labeling ratio of approximately 1.66 ICG molecules per antibody.

### Synthesis of fluorescein isothiocyanate-conjugated antibody (always-on control)

For comparison, we generated an always-on probe (control) by conjugating FITC to Trastuzumab. Trastuzumab (1 mg, 6.8 nmol) was incubated with FITC (67.6 nmol, molar ratio 1:10) in 0.1 mol/L Na_2_HPO_4_ buffer (pH 8.5) at room temperature for 30 minutes. The reaction mixture was purified using a Sephadex G-50 column (PD-10; GE Healthcare). The concentration of FITC was determined by UV–visible absorption spectroscopy, and the degree of labeling was calculated as approximately 1.6 FITC molecules per Trastuzumab molecule.

### *In vitro* quenching assessment

To evaluate the fluorescence quenching capacity of antibody conjugates labeled with ICG or FITC, all conjugates were treated with 1% sodium dodecyl sulfate (SDS) as previously described (24). Briefly, the conjugates were incubated in phosphate-buffered saline (PBS) containing 1% SDS at room temperature for 30 minutes. As a control, parallel samples were incubated in PBS without SDS. Fluorescence intensity was measured using an *in vivo* imaging system (IVIS, PerkinElmer). For ICG-conjugated antibodies, excitation and emission wavelengths were set at 785 nm and 820 nm, respectively. For FITC-conjugated antibodies, excitation and emission wavelengths were set at 485 nm and 520 nm, respectively.

### Cell culture

HER2-positive human breast cancer HCC-1419-Luc cell lines and HER2-negative human breast cancer BT-20-Luc cell lines (JCRB cell bank) were cultured in Dulbecco’s Modified Eagle Medium (DMEM; Nacalai Tesque) and Minimum Essential Medium (MEM; Nacalai Tesque), respectively. Both media were supplemented with 10% fetal bovine serum (FBS; Sigma-Aldrich) and 1% penicillin–streptomycin (Gibco). Cells were incubated at 37°C in a humidified atmosphere containing 5% CO_2_.

### Fluorescence microscopy studies

HCC-1419-Luc and BT-20-Luc cell lines (1 × 10^4^) were seeded into glass-bottom dishes and incubated for 24 hours. Trastuzumab–ICG conjugate (5 μg/mL) was then added to the culture medium and incubated for 1 and 6 hours. In parallel, cells were incubated with a Trastuzumab–FITC conjugate (5 μg/mL) for 30 minutes or 2 hours. After incubation, the cells were washed once with PBS and observed using a BZ-X710 fluorescence microscope (Keyence Corporation, Japan). For Trastuzumab–ICG imaging, the microscope was equipped with filters for excitation at 740–770 nm and emission at 770–800 nm. For Trastuzumab–FITC imaging, filters with excitation at 470–495 nm and emission at 510–550 nm were employed.

### Flow cytometry

HCC-1419-Luc and BT-20-Luc cell lines (1 × 10^5^) were seeded into 35 mm dishes and incubated for 24 hours. Subsequently, a Trastuzumab–ICG conjugate (10 μg/mL) was added to the culture medium and incubated for either 1 or 6 hours. In parallel, Trastuzumab–FITC conjugate was added and incubated for 1 hour. After incubation, fluorescence signals were measured using an Attune NxT flow cytometer (Thermo Fisher Scientific, USA). To confirm the specificity of antibody binding, 100X of unlabeled Trastuzumab was used for blocking studies. Specifically, cells were first incubated with 100X unlabeled Trastuzumab for 30 minutes, followed by incubation with the Trastuzumab–ICG conjugate for an additional 1 hour. The flow cytometry data were analyzed using the free online software Floreada.io (https://floreada.io/).

### *In vitro* fluorescence imaging

HCC-1419-Luc and BT-20-Luc cell lines (1 × 10^4^) were seeded into 24-well black plates and incubated for 24 hours. The Trastuzumab–ICG conjugate (5 μg/mL) was then added to the culture medium, and the cells were further incubated at the specified time points of 30 minutes and 6 hours. Blocking studies with 100X unlabeled Trastuzumab were also performed before adding the Trastuzumab–ICG conjugate. After incubation, cells were washed once with PBS, and fluorescence images were taken using an IVIS Spectrum imaging system (PerkinElmer, USA).

### Animal tumor model

All procedures were approved by the Institutional Animal Care and Use Committee (IACUC) of the Laboratory Animal Resources Centre at the University of Tsukuba. Female athymic nude (nu/nu) mice aged 4–6 weeks were obtained from Japan SLC, Inc. (Shizuoka, Japan).

For the orthotopic breast tumor model, 1 × 10^6^ HCC-1419-Luc (HER2-positive) or BT-20-Luc (HER2-negative) cells were injected into the left and right mammary ducts, respectively, to generate primary tumors. Tumor growth was monitored weekly by bioluminescence imaging (BLI), and palpable or measurable orthotopic tumors typically developed within approximately 4 weeks.

To concurrently evaluate the detection efficiency of Trastuzumab–ICG against lymph node metastasis, which is difficult to model using the mammary fat pad alone due to thin skin and limited mammary tissue volume, we established an auxiliary SLN metastasis model. For this model, 5 × 10^5^ cells in 50 μL suspension were injected subcutaneously into the left and right hind footpads using a 29-gauge needle inserted at a shallow angle (approximately 2–3 mm depth). This approach allows tumor cells to drain through the lymphatic vessels to the ipsilateral inguinal lymph node (groin), mimicking early lymphatic spread. BLI was performed once per week to assess both primary footpad tumor formation and inguinal lymph node involvement, which generally became detectable after 3 weeks.

Thus, each mouse carried both an orthotopic mammary tumor and a footpad-based SLN metastasis model, enabling simultaneous evaluation of the probe’s performance in anatomically distinct tumor sites within the same animal.

### Animal anesthesia and euthanasia

All animal procedures were carried out in accordance with the guidelines of the Animal Ethics Committee of the University of Tsukuba.

For cell injection and all subsequent *in vivo* imaging procedures, including weekly tumor monitoring, BLI, and fluorescence imaging (FLI), mice were anesthetized with isoflurane inhalation (5% for induction and 2.5% for maintenance) delivered via a calibrated vaporizer, allowing rapid induction, fast recovery, and minimal motion during image acquisition. Ketamine/xylazine anesthesia (ketamine 80 mg/kg and xylazine 10 mg/kg, intraperitoneally) was used exclusively for terminal procedures, such as euthanasia and tissue harvesting.

Before ex vivo imaging, mice were euthanized using ketamine/xylazine at three times the standard anesthetic dose, consistent with the AVMA Guidelines for the Euthanasia of Animals (2020). Deep anesthesia prior to euthanasia was confirmed by the absence of the pedal withdrawal reflex. Death was verified by the cessation of heartbeat and respiration and by mucosal pallor.

### *In vivo*/ex-vivo HER2 targeted imaging studies

Trastuzumab–ICG conjugate (50 μg) was injected via the tail vein of tumor-bearing mice. Antibody-based optical probes typically require sufficient time to achieve high tumor accumulation and background clearance, and our preliminary imaging indicated that tumor-to-background contrast was highest around 48 h after injection. Therefore, fluorescence images were acquired at 48 h after injection using the IVIS Spectrum. After taking *in vivo* images, the mice were euthanized, and the tumors, along with lymph nodes, were excised for ex vivo fluorescence imaging.

For quantitative analysis, tumor region of interests (ROIs) were manually drawn based on the visible tumor contour and the corresponding bioluminescence signal to ensure accurate localization. Background ROIs were placed on a tumor-free region of the lower abdomen (or dorsal tissue). For ex vivo imaging, ROIs were drawn to match the outline of each excised tumor or lymph node, and background ROIs were placed on adjacent non-tumorous tissue within the same field of view. Mean fluorescence intensity was extracted from each ROI for subsequent comparisons.

### Statistical analysis

Data are presented as mean ± standard deviation (SD). Statistical analysis was performed using OriginPro 2025 (OriginLab Corporation, Northampton, MA, USA). Comparisons of fluorescence signal intensity between groups were conducted using a two-tailed unpaired Student’s t-test. A p-value of < 0.05 was considered statistically significant.

## Results

### Quenching capability of Trastuzumab–ICG conjugates

**Figure 1:**
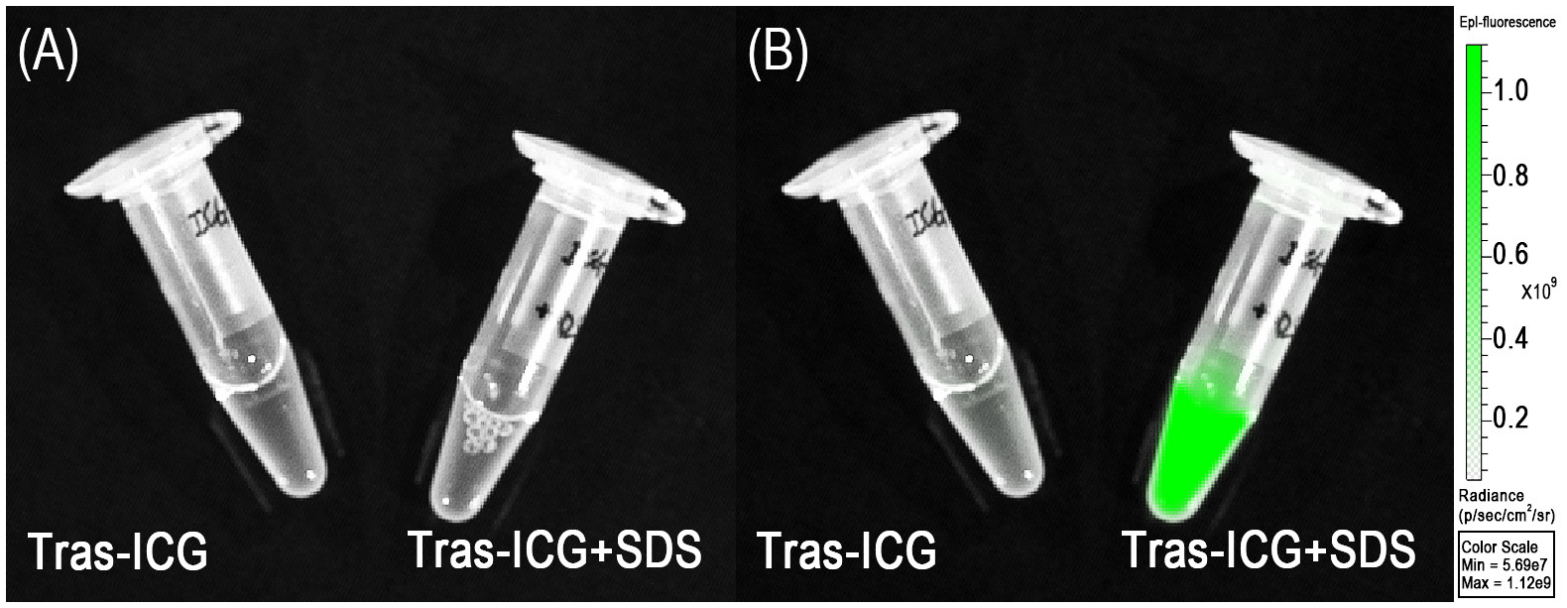
Quenched (left) and chemically activated (right) Trastuzumab–ICG. **(A)** White image. **(B)** Fluorescence image. Activated conjugates exhibited increased fluorescence signals, as indicated by the green color.

**Figure 2:**
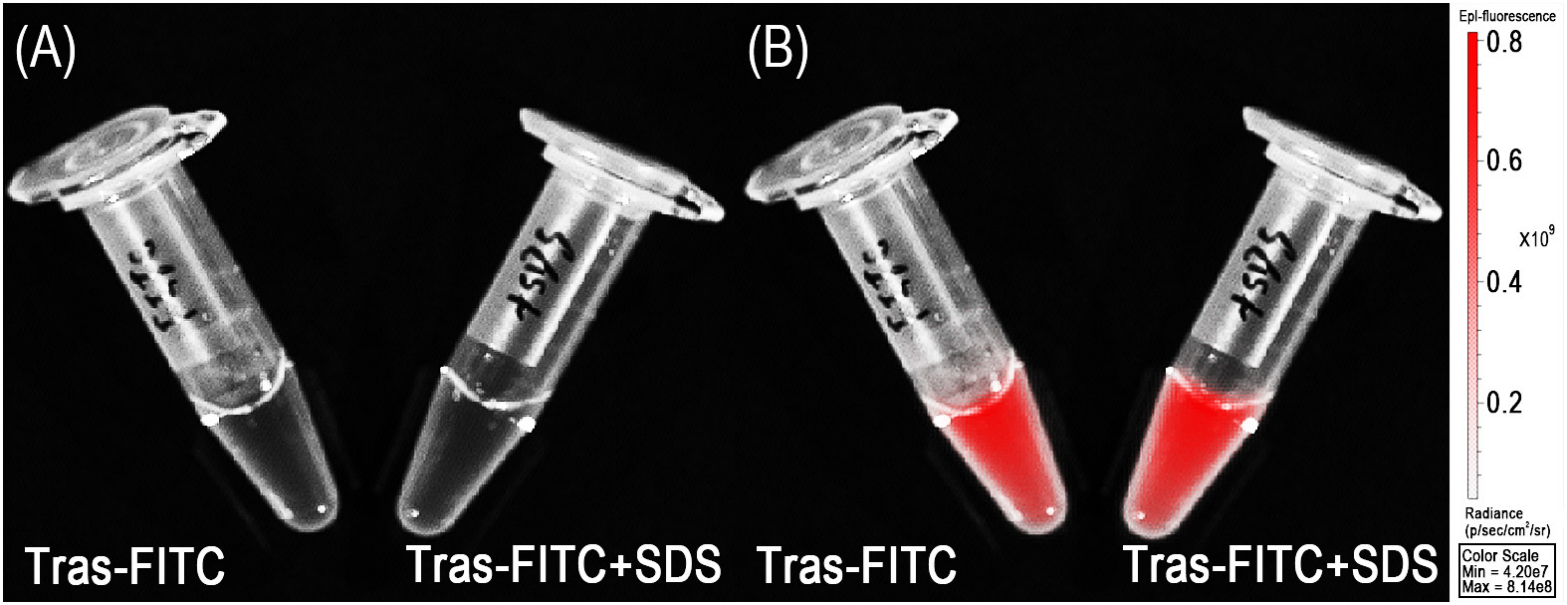
Always-on Trastuzumab–FITC. **(A)** White image. **(B)** Fluorescence image.

The quenching capability was illustrated in **Figure 1**. Before adding SDS, no fluorescence signal was detected in the Trastuzumab–ICG conjugate. However, the fluorescence signal was detected immediately after the addition of SDS. Quantitative analysis confirmed that the fluorescence intensity of the Trastuzumab–ICG conjugate was minimal in the quenched state and increased by approximately 19.6-fold after SDS treatment. In comparison, FITC–antibody conjugates showed fluorescence signals even without the addition of 1% SDS (**Figure 2**).

### *In vitro* fluorescence microscopy

**Figure 3:**
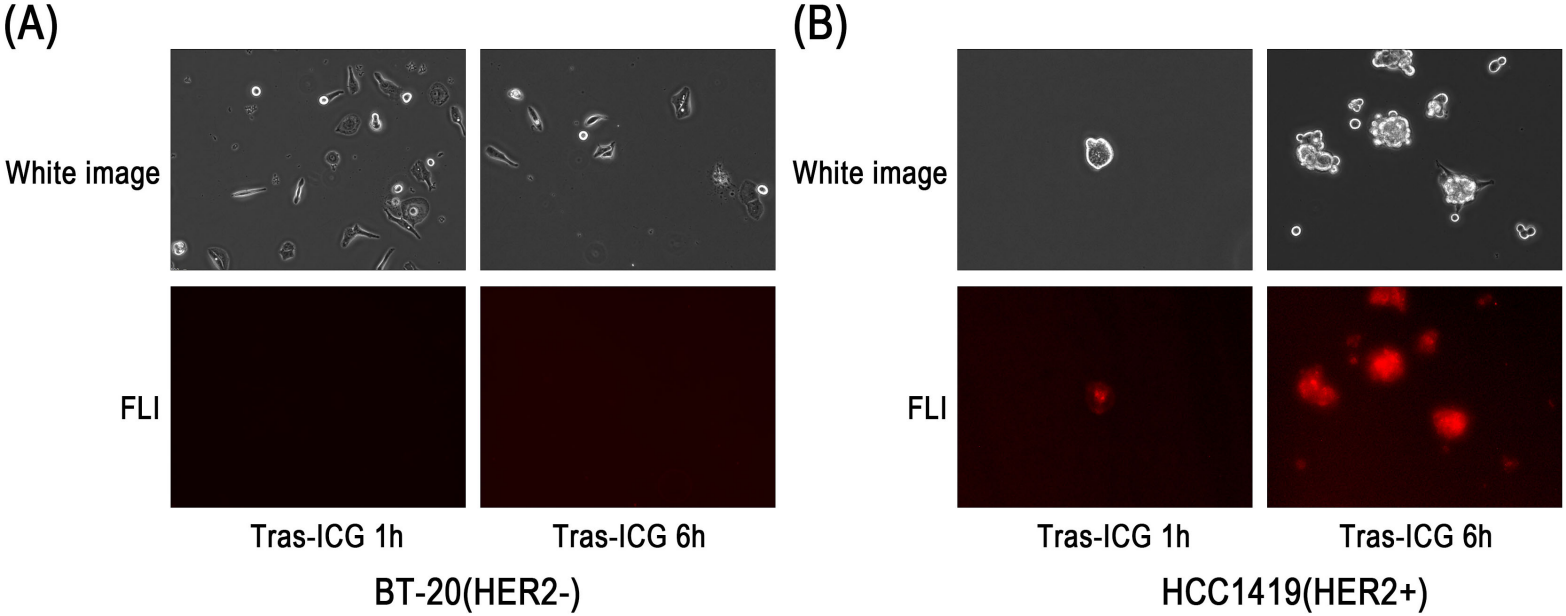
Fluorescence microscopy study of Trastuzumab–ICG binding to BT-20-Luc and HCC-1419-Luc cell lines. After 1 hour (1 h) and 6 hours (6 h) incubation, fluorescence microscopy results showed **(A)** no signal from the HER2-negative cell line (BT-20-Luc) and **(B)** weak signals at 1 h or strong signals at 6 h in the HER2-positive cell line (HCC-1419-Luc).

**Figure 4:**
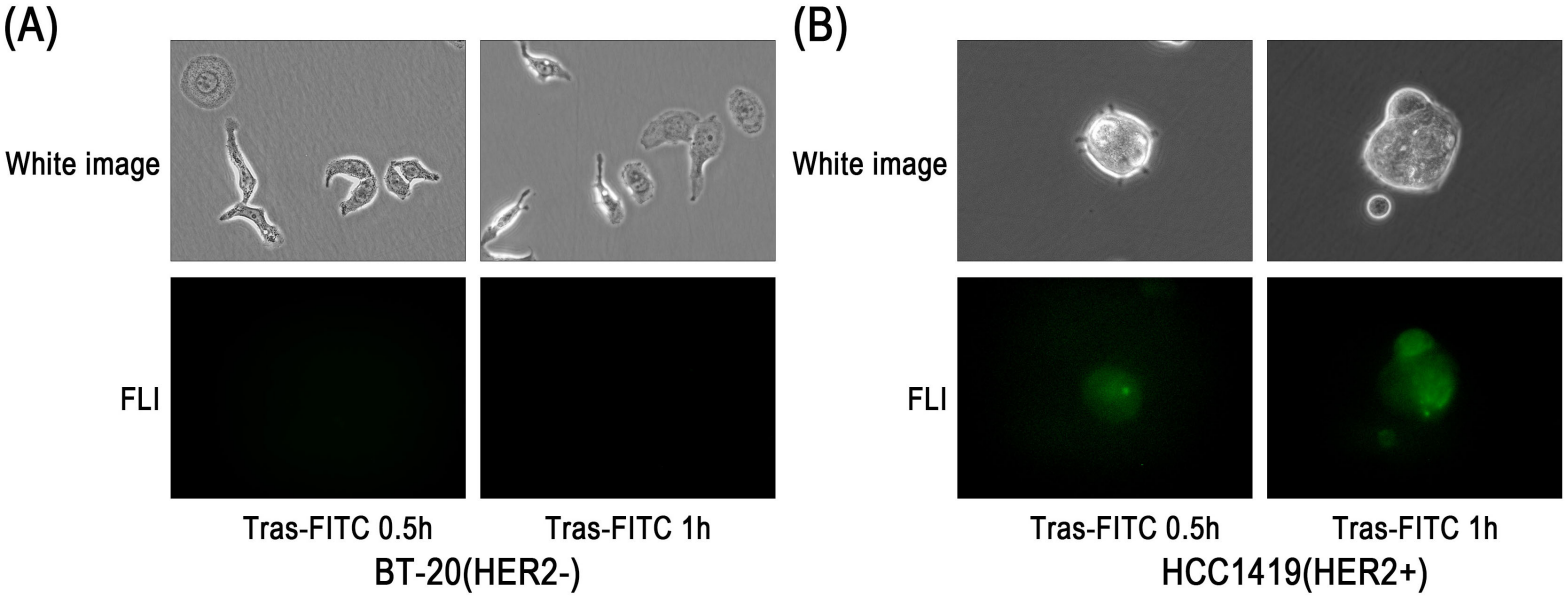
Fluorescence microscopy study of Trastuzumab–FITC binding to BT-20-Luc and HCC-1419-Luc cell lines. After 0.5 h and 1 h incubation, fluorescence microscopy results showed **(A)** weak signals from the HER2-negative cell line (BT-20-Luc) and **(B)** strong signals in the HER2-positive cell line (HCC-1419-Luc).

In HER2-positive HCC-1419-Luc cells, fluorescence signals were observed after 1 and 6 hours of incubation with Trastuzumab–ICG. In contrast, no fluorescence signals were detected in HER2-negative BT-20-Luc cells at either time point (**Figure 3**). Similar results were also observed with the Trastuzumab–FITC conjugate (**Figure 4**).

### *In vitro* fluorescent characterization of probes

**Figure 5:**
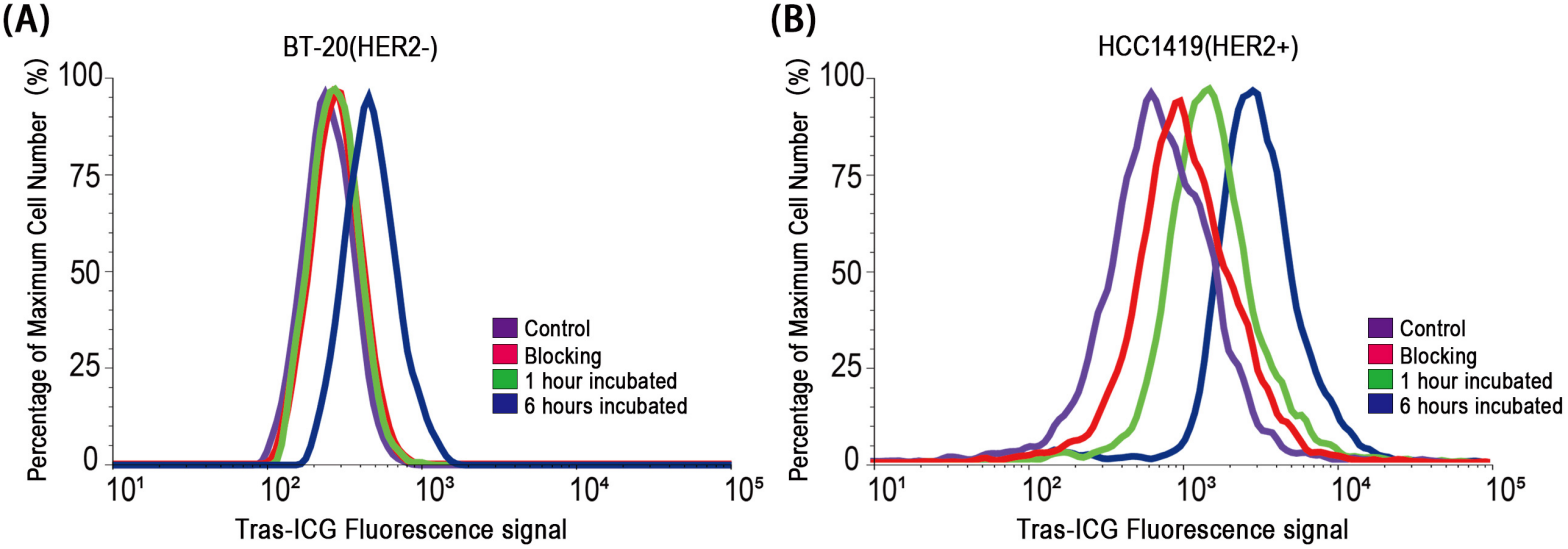
Flow cytometry analysis of BT-20-Luc and HCC-1419-Luc cell lines incubated with Trastuzumab–ICG. *In vitro* experiments showed **(A)** non-specific, weak binding to the HER2-negative cell line at 6 hours and **(B)** specific binding to the HER2-positive cell line at the same point.

Flow cytometry analysis showed a peak shift after 6 hours of incubation with Trastuzumab–ICG in HER2-positive cells, while HER2-negative cells showed only a slight peak shift. This binding was effectively blocked by a 100-fold molar excess of unlabeled Trastuzumab (**Figure 5**).

**Figure 6:**
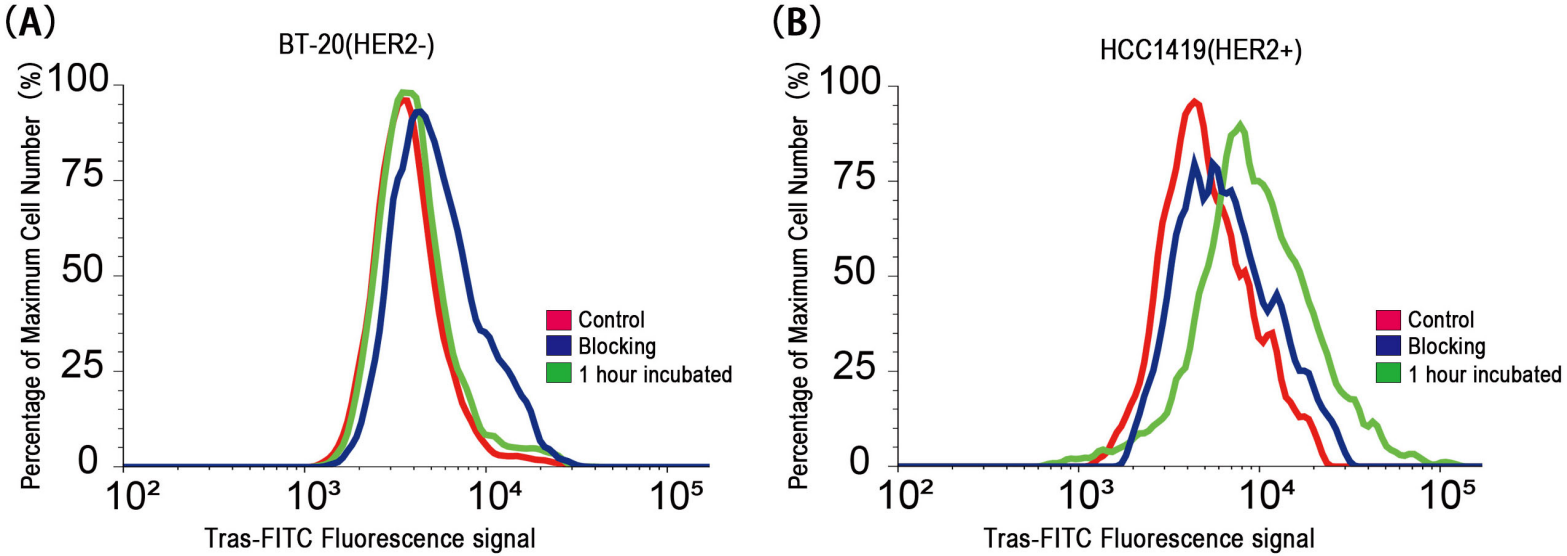
Flow cytometry analysis of BT-20-Luc and HCC-1419-Luc cell lines incubated with Trastuzumab–FITC. *In vitro* experiments showed **(A)** non-specific, weak binding to the HER2-negative cell line and **(B)** specific binding to the HER2-positive cell line.

Flow cytometry analysis using Trastuzumab–FITC demonstrated strong fluorescence signals in HER2-positive cells after 1 hour of incubation, whereas HER2-negative cells showed no significant change (**Figure 6**).

### *In vivo* and ex vivo imaging of HER2-targeted probe

#### Orthotopic tumor model

**Figure 7:**
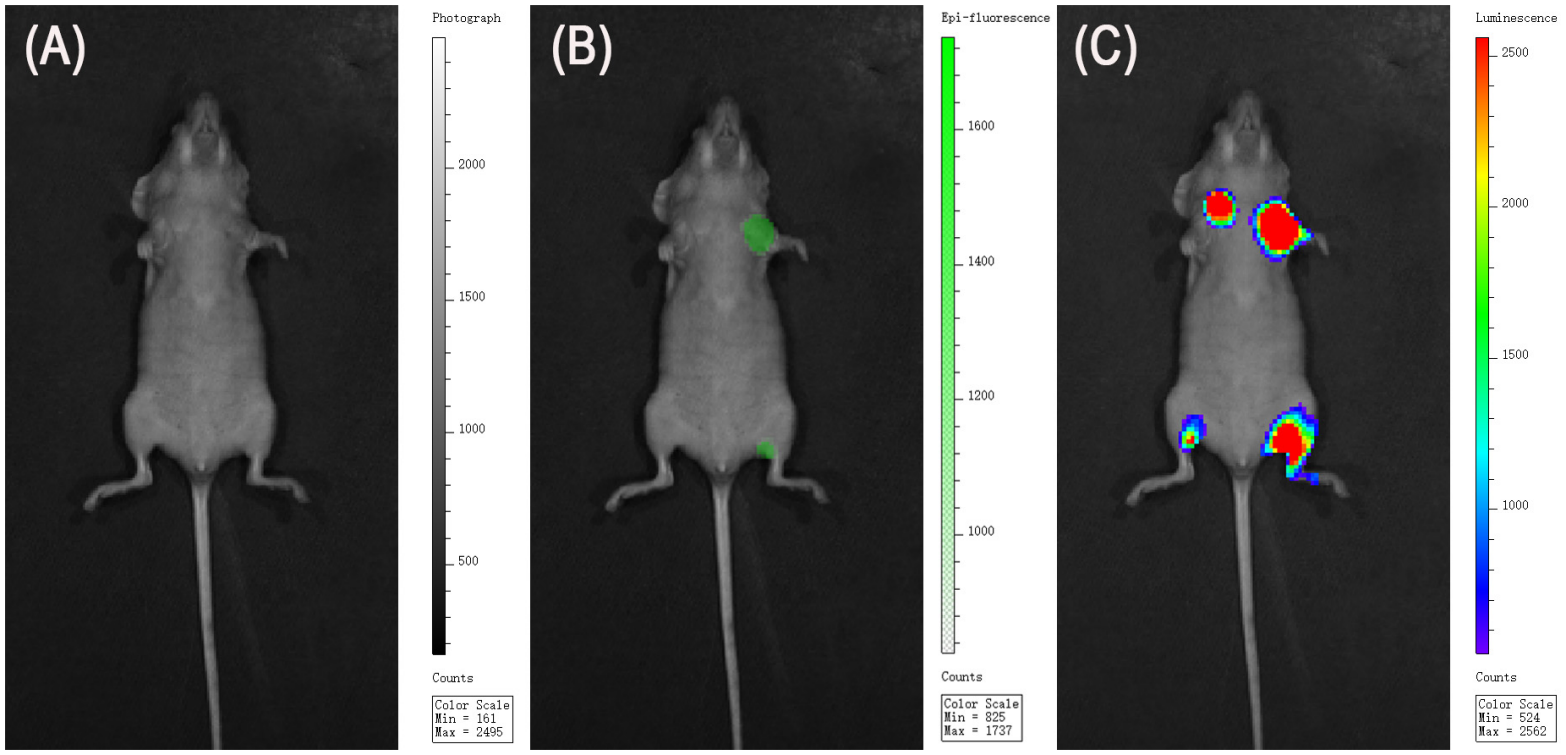
In vivo HER2-targeted imaging. **(A)** White image. **(B)** FLI was used to evaluate the binding of Trastuzumab–ICG. HER2-positive primary tumors and lymph node metastases exhibited higher fluorescence than HER2-negative lesions, consistent with activation of the quenched probe in HER2-positive tissues. **(C)** BLI confirmed tumor formation .In vivo imaging was performed in n = 5 mice, each bearing one HER2-positive and one HER2-negative tumor with their corresponding lymph nodes.

HCC-1419-Luc and BT-20-Luc cells were injected into the left and right mammary ducts of nude mice, respectively. Tumor progression was monitored using bioluminescence imaging (BLI), and successful tumor establishment was confirmed three weeks after implantation (**Figure 7**). Fluorescence imaging performed 48 hours after intravenous administration of Trastuzumab–ICG revealed strong fluorescence signals at the tumor site in HCC-1419-Luc tumors, whereas no apparent fluorescence was observed in BT-20-Luc tumors.

#### SLN metastasis model

In the SLN metastasis model, fluorescence signals were observed in lymph nodes corresponding to the HCC-1419-Luc injection site, while no detectable fluorescence was found in lymph nodes associated with BT-20-Luc cell injection.

**Figure 8:**
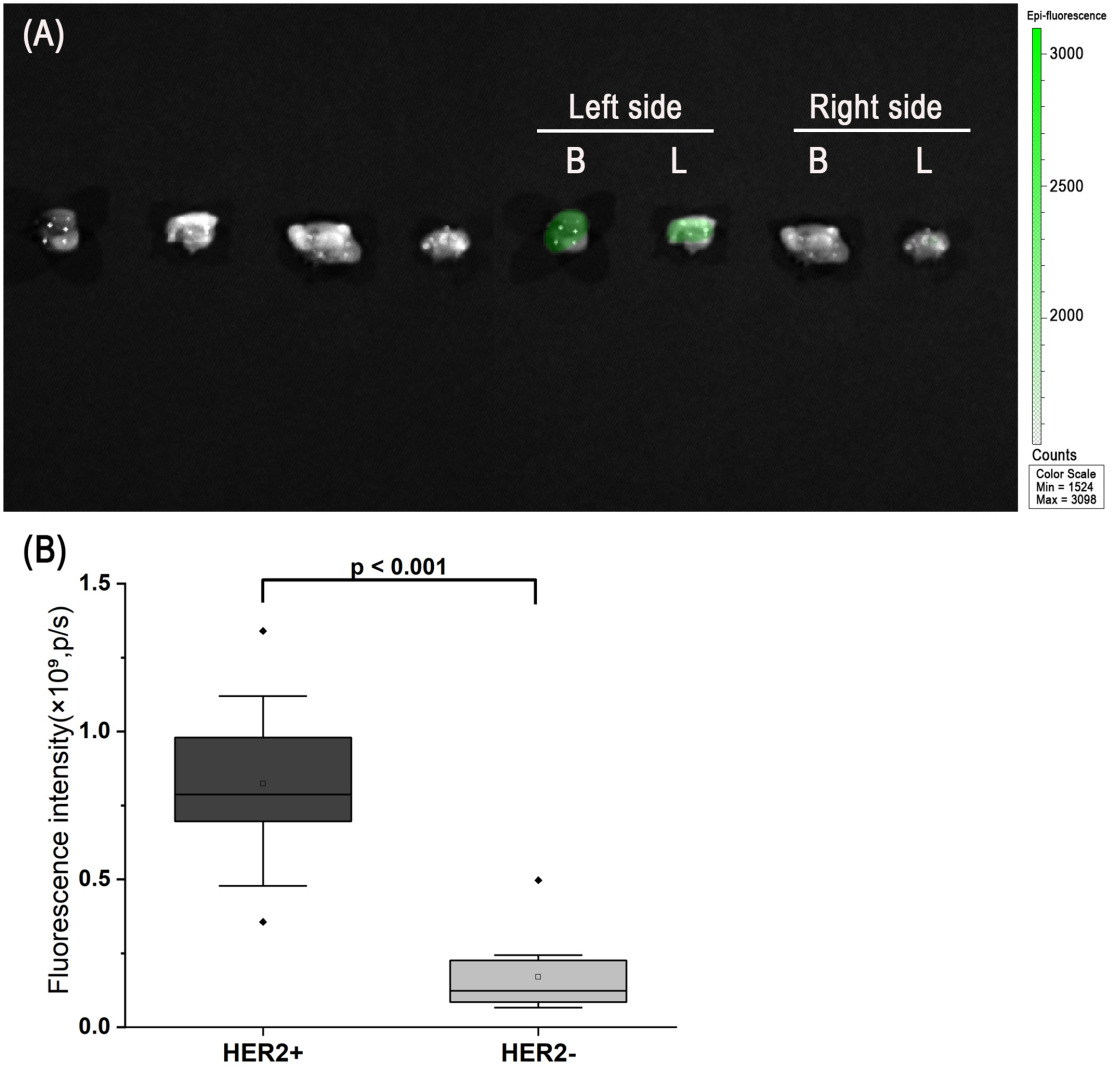
Ex vivo imaging and quantitative analysis of excised tumors (Primary orthotopic breast tumors and lymph node metastatic lesions). **(A)** White light image (left) and fluorescence/white light fusion image (right) of representative tumors. The HER2-positive tumor exhibits a distinct fluorescent signal, whereas the HER2-negative tumor shows no obvious signal. **(B)** Quantitative fluorescence intensity of the tumors shown in **(A)**, demonstrating significantly higher signal in the HER2-positive group (p < 0.001). Plotted values represent raw fluorescence ROI intensities. In total, n = 10 HER2-positive tumors and n = 10 HER2-negative tumors were analyzed.

**Figure 9:**
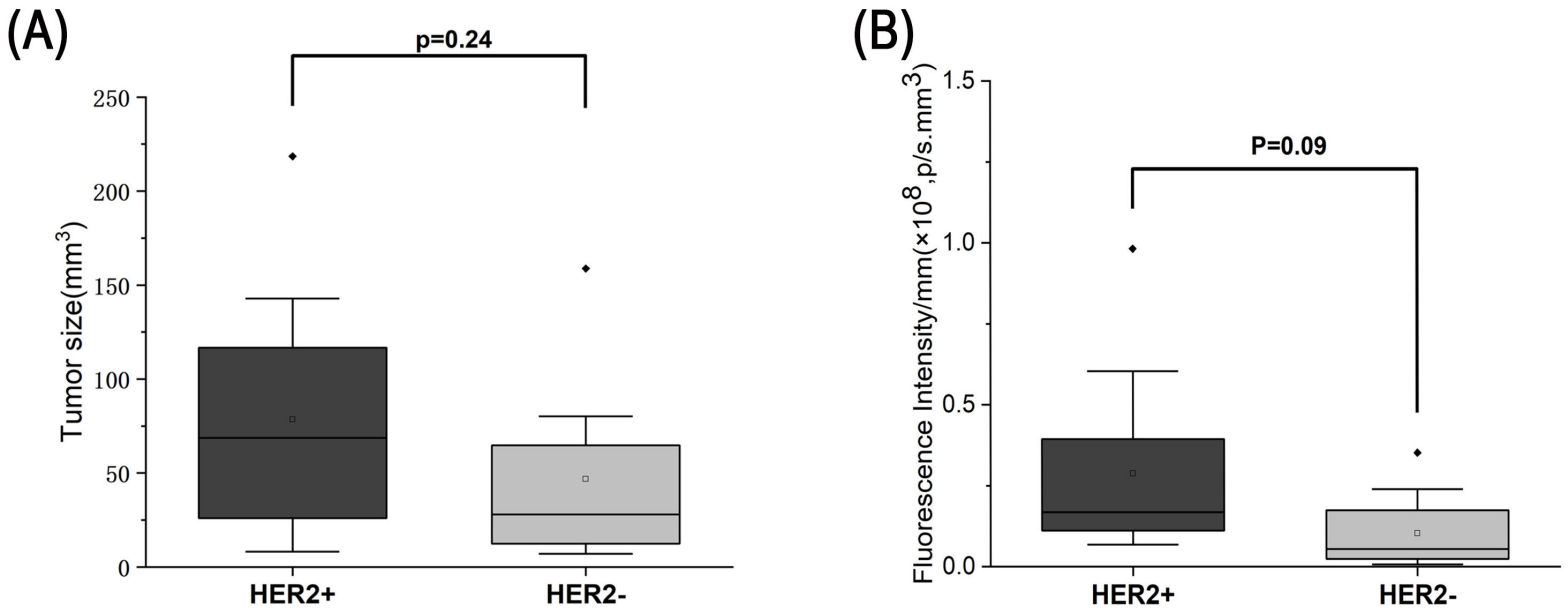
Comparison between tumors derived from HER2-positive and HER2-negative cells. **(A)** Comparison of tumor size between groups revealed no significant difference. **(B)** Quantification of bioluminescence signal normalized by tumor volume; no statistically significant difference was detected. Plotted BLI values are normalized (radiance per mm^3^). Analyses were performed on n = 10 HER2-positive tumors and n = 10 HER2-negative tumors.

Following *in vivo* imaging, tumors and lymph nodes were excised for ex vivo fluorescence imaging. Ex vivo imaging of primary orthotopic tumors showed a distinct fluorescent signal in HER2-positive tumors, while HER2-negative tumors showed minimal to no signal (**Figure 8A**). Quantitative analysis demonstrated significantly higher fluorescence intensity in HER2-positive tumors compared with HER2-negative tumors (**Figure 8B**, p < 0.001). Consistent with this, ex vivo lymph node imaging also revealed higher fluorescence intensity in HER2-positive metastatic lymph nodes compared with HER2-negative nodes. No significant difference in tumor size was observed between the HER2-positive and HER2-negative groups (**Figure 9**).

## Discussion

In this study, we demonstrated that the Trastuzumab–ICG activatable probe enabled precise fluorescence detection of HER2-positive breast cancer cells and tumors both *in vitro* and *in vivo*. The probe remained quenched during circulation, minimizing background signals, and was activated only after binding to HER2 and subsequent lysosomal degradation. This mechanism produced a high tumor-to-background contrast in both orthotopic tumors and SLN metastasis models (25–27). Control experiments with Trastuzumab–FITC confirmed the superiority of the activatable design, as fluorescence was restricted to HER2-positive cells while negligible in HER2-negative cells. Furthermore, flow cytometry blocking studies using a 100-fold molar excess of unlabeled Trastuzumab effectively inhibited nonspecific binding, validating the high specificity of Trastuzumab–ICG accumulation.

The strength of this probe lies in its activatable quenched mechanism. By placing multiple ICG molecules in proximity to the antibody, self-quenching occurs through FRET or aggregation during circulation, effectively suppressing background fluorescence (25–28). Fluorescence is restored only upon binding to HER2, followed by cellular uptake and lysosomal degradation, which separates the fluorophores from the antibody scaffold. This on-off activation ensures that signals originate exclusively from HER2-expressing tumor cells while surrounding tissues remain dark. In contrast, always-on probes, such as Trastuzumab–FITC, constantly emit fluorescence regardless of cellular context, leading to higher background and lower imaging contrast. The activatable design, therefore, provides a critical advantage for intraoperative imaging, where high tumor-to-background ratios are essential (28).

Although free ICG is often injected peritumorally in clinical SLN mapping, antibody-based probes such as Trastuzumab–ICG require intravenous administration to circulate systemically, engage HER2 receptors, undergo internalization, and achieve lysosomal activation. Peritumoral injection could theoretically deliver the probe locally, but restrict its distribution at the injection site, limiting access to HER2-positive tumors or metastatic lymph nodes. Therefore, while feasible, peritumoral delivery would not achieve the intended target-specific activation or reflect the translational use of an antibody-based activatable probe.

Several HER2-targeted NIR imaging agents have been evaluated in preclinical models and early clinical studies, including Trastuzumab-based probes conjugated with IRDye800CW and affibody-derived HER2 tracers (29, 30). These agents demonstrated the feasibility of optical visualization of HER2-positive breast cancer; however, they typically rely on constitutively fluorescent (‘always-on’) designs, which may produce suboptimal tumor-to-background contrast due to circulating probe or nonspecific uptake (31). In contrast, activatable ‘off–on’ probes such as our Trastuzumab–ICG conjugate remain quenched during systemic circulation and become fluorescent only upon target engagement and intracellular processing. This design substantially enhances tumor specificity and improves tumor-to-background ratios, reinforcing the translational potential of HER2-targeted fluorescence imaging for intraoperative use.

Accurate intraoperative detection of SLN metastasis is vital for staging and surgical planning (32, 33). Current approaches, including frozen section analysis and OSNA, are widely used but require invasive lymph node excision, are time-consuming, and rely heavily on technical expertise (34–36). They also carry risks of sampling error and delays in intraoperative decision-making. In contrast, the Trastuzumab–ICG probe provides non-invasive and immediate visualization of HER2-positive tumors and lymph node metastases directly within the surgical field. This capacity for instant visualization offers surgeons direct feedback, potentially reducing unnecessary axillary dissections, improving staging accuracy, and facilitating personalized surgical strategies. Furthermore, it may complement existing pathological methods by reducing waiting times and providing rapid confirmation of metastatic burden in complex intraoperative cases. Per-mouse dose of Trastuzumab delivered by the probe was approximately 20 μg (≈1 mg/kg for a 20 g mouse), which is far below the therapeutic dose typically required for anti-tumor activity (commonly ≥20–30 mg/kg in mouse models and ≈4 mg/kg clinically). Moreover, preclinical studies have shown that even mg/kg-level Trastuzumab monotherapy often has little or no measurable impact on tumor growth in aggressive HER2-driven models (37, 38), therefore, in our study, the tumor growth was not affected by probe administration.

Although both Trastuzumab and ICG are clinically approved components, the safety profile of the conjugated activatable probe, including potential toxicity, immunogenicity, and *in vivo* stability require further systematic evaluation. Also, pharmacokinetic and dose-optimization studies are needed to refine the imaging window and minimize off-target accumulation. In addition, assessing compatibility with clinically available NIR imaging systems used in fluorescence-guided surgery will be essential to ensure seamless intraoperative implementation. These will support the progression of this activatable HER2-targeted probe from the present proof-of-concept stage toward potential clinical application.

## Limitations

Several limitations must be acknowledged. First, the use of immunodeficient mouse models does not fully recapitulate the complex interactions between the immune system, tumor microenvironment, and antibody-based imaging probes. Second, although representative HER2-positive and HER2-negative breast cancer cell lines were used, validation across a broader panel of molecular subtypes will be necessary to confirm generalizability. Third, although lymph node involvement modeled using footpad injection enables reproducible assessment of nodal metastasis, it does not fully replicate spontaneous axillary dissemination observed in patients. Fourth, Trastuzumab-ICG is inherently limited to HER2-positive tumors, which account for only 20–25% of breast cancers (39–41).

Despite these limitations, activatable antibody–fluorophore conjugates hold considerable promise as next-generation tools for fluorescence image-guided surgery (FIGS). Extending this concept to other tumor-associated antigens could broaden applicability to different breast cancer subtypes and other malignancies. Additionally, integration with hybrid modalities, such as photoacoustic or PET imaging, could provide comprehensive intraoperative tumor mapping. Advances in AI-assisted intraoperative analysis may further enhance the interpretation of fluorescence signals, supporting real-time surgical decision-making. Collectively, these innovations may transform activatable fluorescence probes into powerful tools for precision oncology, ultimately enhancing the accuracy of cancer surgery and improving patient outcomes.

## Data Availability

The original contributions presented in the study are included in the article/supplementary material. Further inquiries can be directed to the corresponding author.
